# Green tea extract prevents CPT-11-induced diarrhea by regulating the gut microbiota

**DOI:** 10.1038/s41598-023-33731-w

**Published:** 2023-04-21

**Authors:** Risako Kon, Nobutomo Ikarashi, Arisa Yamaguchi, Yuka Teshima, Tamami Yamaguchi, Kanako Miyaoka, Moeno Fukuda, Hinata Noguchi, Rei Tomimoto, Hiroyasu Sakai, Junzo Kamei, Tomoo Hosoe

**Affiliations:** 1 Department of Biomolecular Pharmacology, Hoshi University, 2-4-41 Ebara, Shinagawa-ku, Tokyo, 142-8501 Japan; 2 Juntendo Advanced Research Institute for Health Science, Juntendo University, 2-4-4 Hongo, Bunkyo-ku, Tokyo, 113-8421 Japan

**Keywords:** Health care, Medical research

## Abstract

Irinotecan (CPT-11) is an anticancer drug with indications for use in treating various cancers, but severe diarrhea develops as a side effect. We investigated the effects of green tea extract (GTE) on CPT-11-induced diarrhea, focusing on β-glucuronidase and intestinal UGT1A1. When CPT-11 was administered to rats alone, the fecal water content was approximately 3.5-fold higher in this group than in the control group, and diarrhea developed. The fecal water content in the GTE-treated group was significantly higher than that in the control group, but the difference was smaller than that between the group treated with CPT-11 alone and the control group, and diarrhea improved. When CPT-11 was administered alone, the abundances of *Bacteroides* *fragilis* and *Escherichia* *coli*, which are β-glucuronidase-producing bacteria, increased and interleukin-6 and interleukin-1β mRNA levels in the colon increased, but GTE suppressed these increases. CPT-11 decreased colon UGT1A1 and short-chain fatty acid levels; however, this decrease was suppressed in the GTE-treated group. The findings that GTE decreases the abundance of β-glucuronidase-producing bacteria and increases colon UGT1A1 levels, thereby decreasing the production of the active metabolite SN-38 in the intestinal tract, indicate that GTE ameliorates CPT-11-induced diarrhea.

## Introduction

Irinotecan (CPT-11), an anticancer drug, is effective against various cancers. However, gastrointestinal disorders, such as diarrhea, anorexia, stomatitis, nausea, and vomiting, frequently occur during the use of CPT-11. In particular, diarrhea is a side effect observed in approximately 80% of patients and is a factor that considerably reduces the quality of life (QOL)^1,2^.

CPT-11-induced diarrhea is classified into early-onset and late-onset^3^. Early-onset diarrhea results from the inhibition of acetylcholinesterase, which activates the parasympathetic nerves, resulting in increased intestinal peristalsis. Therefore, this early-onset diarrhea is ameliorated by the administration of anticholinergic drugs such as atropine^4,5^. However, late-onset diarrhea is extremely serious and results from the following mechanism. CPT-11 is first metabolized to the active metabolite 7-ethyl-10-hydroxycamptothecin (SN-38) by carboxyesterases in the liver. SN-38 undergoes glucuronidation by uridine diphosphate-glucuronosyltransferase 1A1 (UGT1A1) to form SN-38-glucuronide (SN-38G), which is excreted in bile and transported into the intestinal tract. SN-38G is deconjugated by β-glucuronidase derived from intestinal bacteria and converted to SN-38 again^6–8^. SN-38 causes mucosal damage in the intestinal tract, resulting in severe diarrhea as a side effect^9^. At present, loperamide, which inhibits intestinal peristalsis, and hange-shashinto (HST), a traditional Kampo medicine that inhibits β-glucuronidase, are used to treat CPT-11-induced late-onset diarrhea^10,11^. However, the therapeutic effects of these agents are not sufficient, and in some patients with severe symptoms, the dosage of CPT-11 is reduced or the drug is discontinued. There are reports that cyclooxygenase-2 inhibitors, antibacterial drugs, and curcumin are also useful in the treatment of diarrhea^12–14^, but their effects are limited, and they have not yet reached clinical application. Therefore, the discovery of new preventive and therapeutic agents for CPT-11-induced diarrhea is extremely important for cancer treatment.

**Figure 1:**
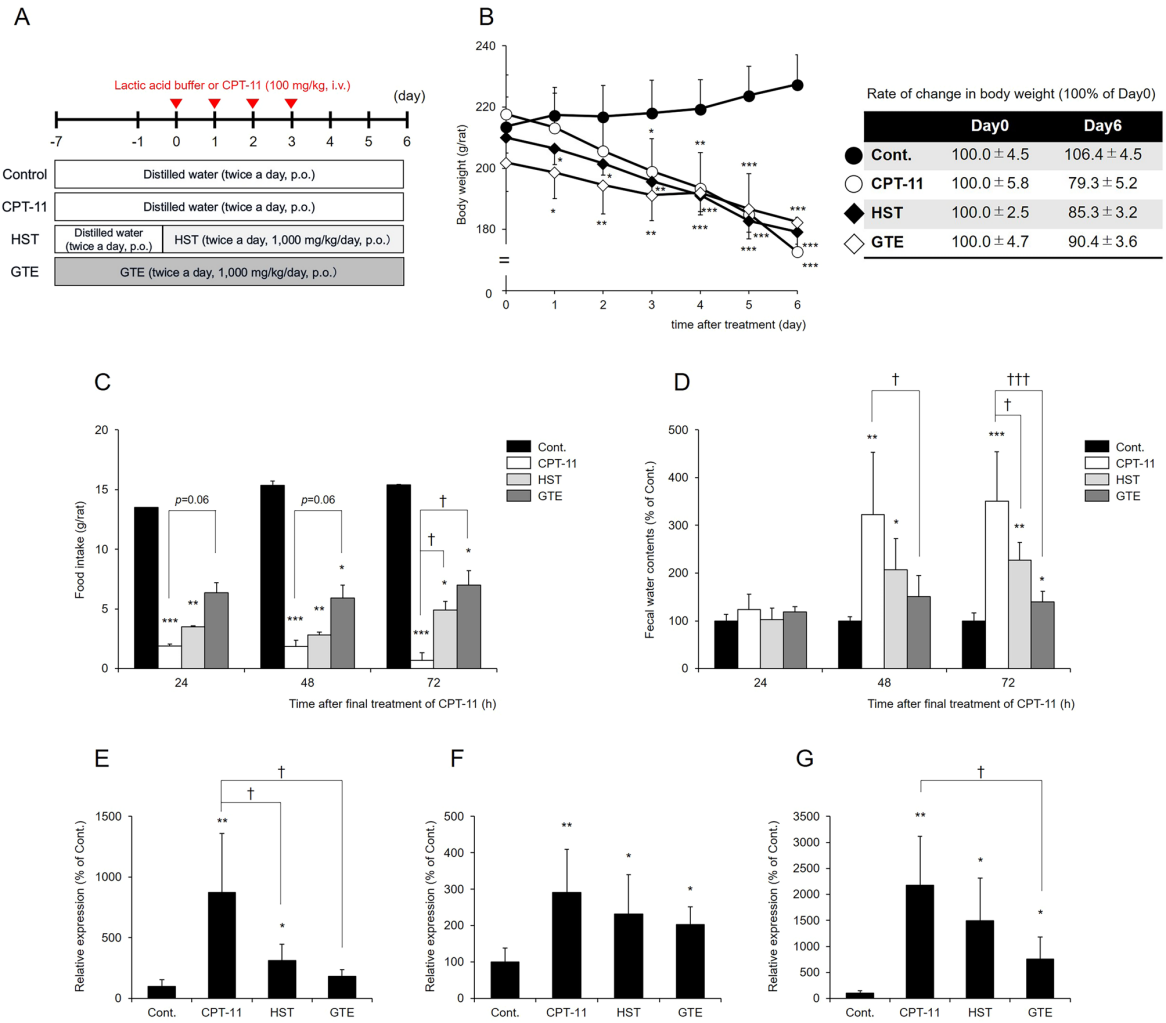
Effects of GTE on CPT-11-induced delayed diarrhea. Rats were administered CPT-11 or lactic acid buffer intravenously for 4 days, and HST or GTE was administered in combination (**A**). Body weight (**B**), 24-h food intake (**C**), and fecal water content (**D**) were measured. The water content in feces is shown as 100% of the mean value in the control group. The mRNA levels of IL-6 (**E**), IL-1β (**F**), and iNOS (**G**) in the colon were measured by real-time PCR. The levels of each gene were normalized using β-actin levels and are shown as 100% of the mean value in the control group (mean ± S.D., n = 5, *p < 0.05, **p < 0.01, *** *p* < 0.001 vs. Cont., ^†^*p* < 0.05, ^†††^*p* < 0.001 vs. CPT-11).

It was recently reported that polyphenols contained in the leaves of *Camellia* *sinensis* inhibit β-glucuronidase activity^15^. Processed leaves are consumed as green tea and other luxury items, and the extracted ingredients are used as health care supplements. Based on these findings, we hypothesized that the use of beverages or supplements containing green tea polyphenols during cancer chemotherapy may prevent CPT-11-induced diarrhea. In this study, we focused on green tea extract (GTE) as a new tool to prevent CPT-11-induced late-onset diarrhea and investigated its usefulness. Specifically, we examined the degree of diarrhea in a rat model of CPT-11-induced late-onset (delayed) diarrhea when GTE was administered in combination with CPT-11 (Fig. 1A). In this study, HST, which has been reported to suppress CPT-11-induced diarrhea, was used as a positive control to compare its effects^16^.

## Results

### Effects of GTE on CPT-11-induced delayed diarrhea

The body weight of rats in the group treated with CPT-11 alone began to decrease from the second day of CPT-11 administration, and on the final day, it was approximately 80% of that on Day 0. In contrast, the body weight in the GTE-treated group was significantly lower than that in the control group, but the change was milder than that in the CPT-11 alone group (Fig. 1B). Food intake by rats treated with CPT-11 alone significantly decreased to approximately 5% of that in the control group at 72 h after the last CPT-11 administration. In the GTE-treated group, the food intake was lower than that in the control group, but it was significantly higher than that in the group treated with CPT-11 alone, and constant food intake was maintained during the period (Fig. 1C). These results indicated that GTE suppressed the CPT-11-induced decrease in body weight and appetite, and this effect tended to be stronger than that of HST.

In the group treated with CPT-11 alone, the fecal water content began to increase 48 h after the final CPT-11 administration and was 3.5 times higher than that in the control group at 72 h, and diarrhea developed (Fig. 1D). At this time, the mRNA levels of interleukin-6 (IL-6), interleukin-1β (IL-1β), and inducible nitric oxide synthase (iNOS) increased in the colon, and inflammation occurred (Fig. 1E–G). In the GTE-treated group, the fecal water content was significantly lower than that in the group treated with CPT-11 alone during the onset of diarrhea and was significantly lower than that in the HST-treated group after 72 h (*p* = 0.002 vs. HST) (Fig. 1D). In addition, the expression levels of IL-6 and iNOS in the GTE-treated group were significantly lower than those in the CPT-11 alone group (Fig. 1E,G). These results indicated that GTE suppressed CPT-11-induced diarrhea and colonic inflammation.

### Effects of GTE on β-glucuronidase activity and β-glucuronidase-producing bacteria

**Figure 2:**
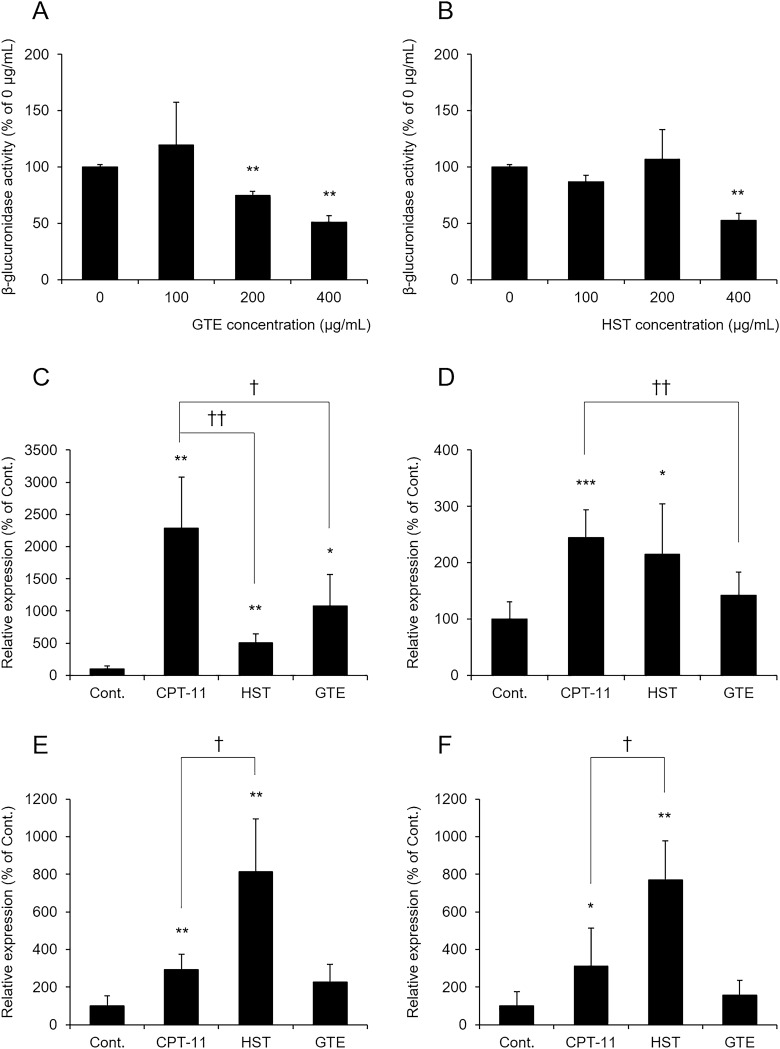
Effects of GTE on β-glucuronidase activity and bacteria involved in β-glucuronidase production. (**A,B**) GTE (**A**) or HST (**B**) was mixed with enzyme solution extracted from rat feces, and β-glucuronidase activity was evaluated. The mean value in the control group (0 μg/mL) is shown as 100% (mean ± S.D., n = 3, **p < 0.01 vs. 0 μg/mL). (**C–F**) Rats were administered CPT-11 or lactic acid buffer intravenously for 4 days, and HST or GTE was administered in combination. The levels of *B.* *fragilis* (**C**), *E.* *coli* (**D**), *C.* *perfringens* (**E**), and *E.* *eligens* (**F**) were measured by real-time PCR. Each value was normalized using 16S rRNA levels and is shown as 100% of the mean value in the control group (mean ± S.D., n = 5, *p < 0.05, **p < 0.01, ***p < 0.001 vs. Cont., ^†^*p* < 0.05, ^††^*p* < 0.01 vs. CPT-11).

CPT-11-induced diarrhea is caused by SN-38, an active metabolite produced by β-glucuronidase derived from intestinal bacteria^8^. Therefore, we investigated whether GTE directly inhibits β-glucuronidase activity by an in vitro study. The results showed that GTE decreased β-glucuronidase activity in a dose-dependent manner (Fig. 2A). In contrast, HST inhibited β-glucuronidase activity only at a concentration of 400 μg/mL (Fig. 2B). These results indicated that GTE directly inhibited β-glucuronidase activity, and this effect was stronger than that of HST.

In the CPT-11 alone group, the levels of *Bacteroides* *fragilis*, *Escherichia* *coli*, *Clostridium* *perfringens*, and *Eubacterium* *eligens* producing β-glucuronidase with high SN-38G deconjugation activity^17^ were all significantly increased compared to those in the control group (Fig. 2C–F). In the GTE-treated group, the increase in *B.* *fragilis* and *E.* *coli* abundances induced by CPT-11 was significantly suppressed. Although HST suppressed the increase in *B.* *fragilis* abundance induced by CPT-11, it further enhanced the CPT-11-induced increase in *C.* *perfringens* and *E.* *eligens* abundances. These results indicated that GTE regulated the abundances of β-glucuronidase-producing bacteria.

### Effects of GTE on gastrointestinal UGT1A1

According to previous reports, CPT-11-induced delayed diarrhea is more severe in patients with UGT1A1 gene polymorphisms^18^. Briefly, this is because SN-38 directly enters the intestinal tract as a result of decreased UGT1A1 activity in the liver. UGT1A1 is also highly expressed in the gastrointestinal tract^19,20^. Recent papers showed that CPT-11-induced gastrointestinal inflammation and mucosal damage were lower in mice that experimentally expressed UGT1A1 specifically in the gut than in mice with liver-specific expression^21^. Therefore, we focused on gastrointestinal UGT1A1 as a mechanism by which GTE improves CPT-11-induced delayed diarrhea.

**Figure 3:**
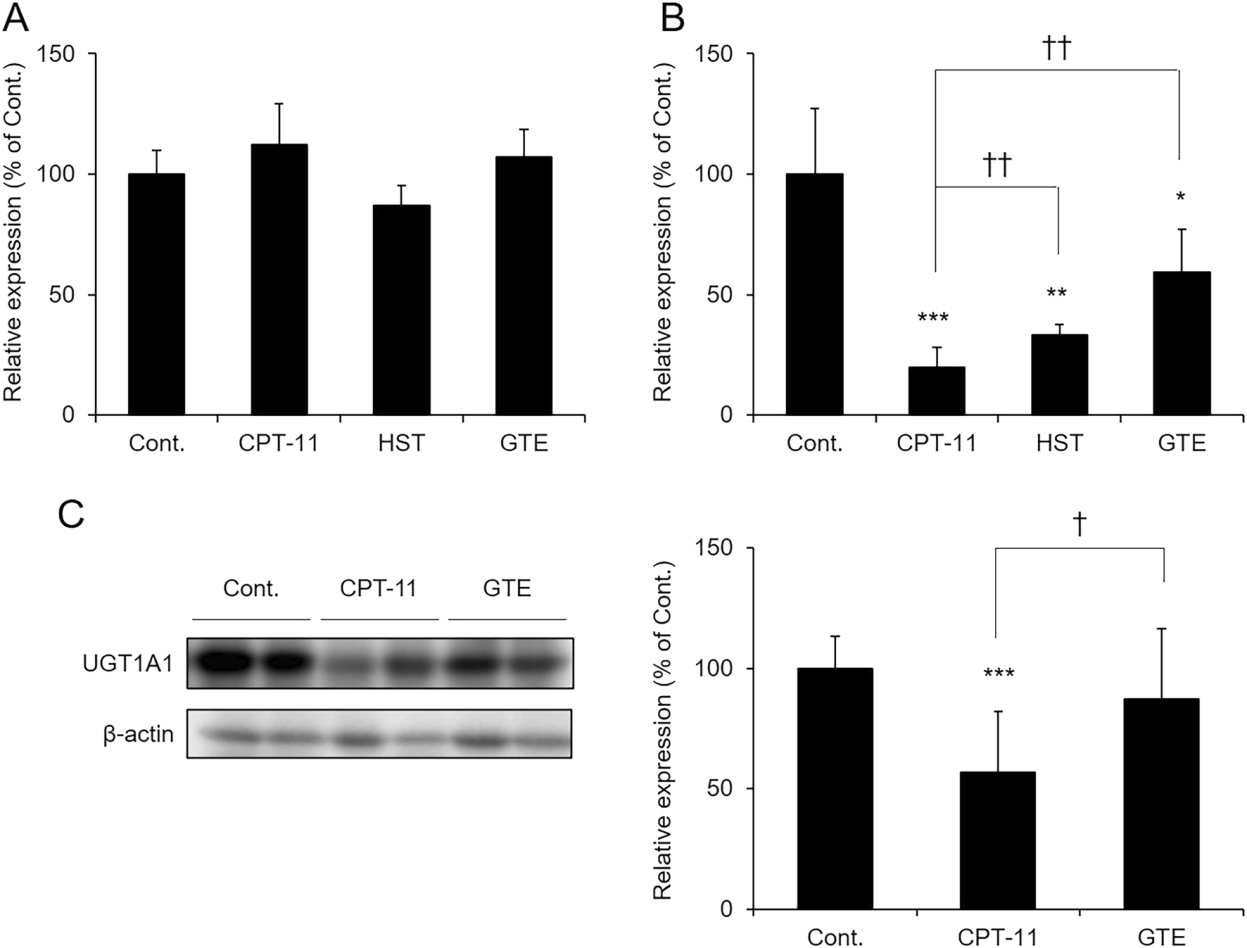
Changes in intestinal UGT1A1 expression after CPT-11 and GTE treatment. Rats were administered CPT-11 or lactic acid buffer intravenously for 4 days, and HST or GTE was administered in combination. The mRNA and protein expression levels of UGT1A1 in the small intestine (**A**) or colon (**B** or **C**) were analyzed by real-time PCR or Western blotting. Each value was normalized using β-actin levels and is shown as 100% of the mean value in the control group (mean ± S.D., n = 5, *p < 0.05, **p < 0.01, ***p < 0.001 vs. Cont., ^†^*p* < 0.05, ^††^*p* < 0.01 vs. CPT-11).

CPT-11 treatment alone did not alter UGT1A1 mRNA expression in the rat ileum. This finding was similarly observed in rats administered GTE or HST (Fig. 3A). However, the mRNA and protein levels of UGT1A1 in the colon were significantly lower in the group administered CPT-11 alone than in the control group, but the decrease was suppressed in the GTE-treated group. Although the CPT-11-induced decrease in colonic UGT1A1 expression was inhibited in the HST-treated group, this effect was weaker than that observed in the GTE-treated group (p = 0.03 vs. GTE) (Fig. 3B, C). These results indicated that GTE suppressed the decrease in colonic UGT1A1 expression induced by CPT-11.

### Effects of GTE on UGT1A1 expression levels in HT-29 cells

**Figure 4:**
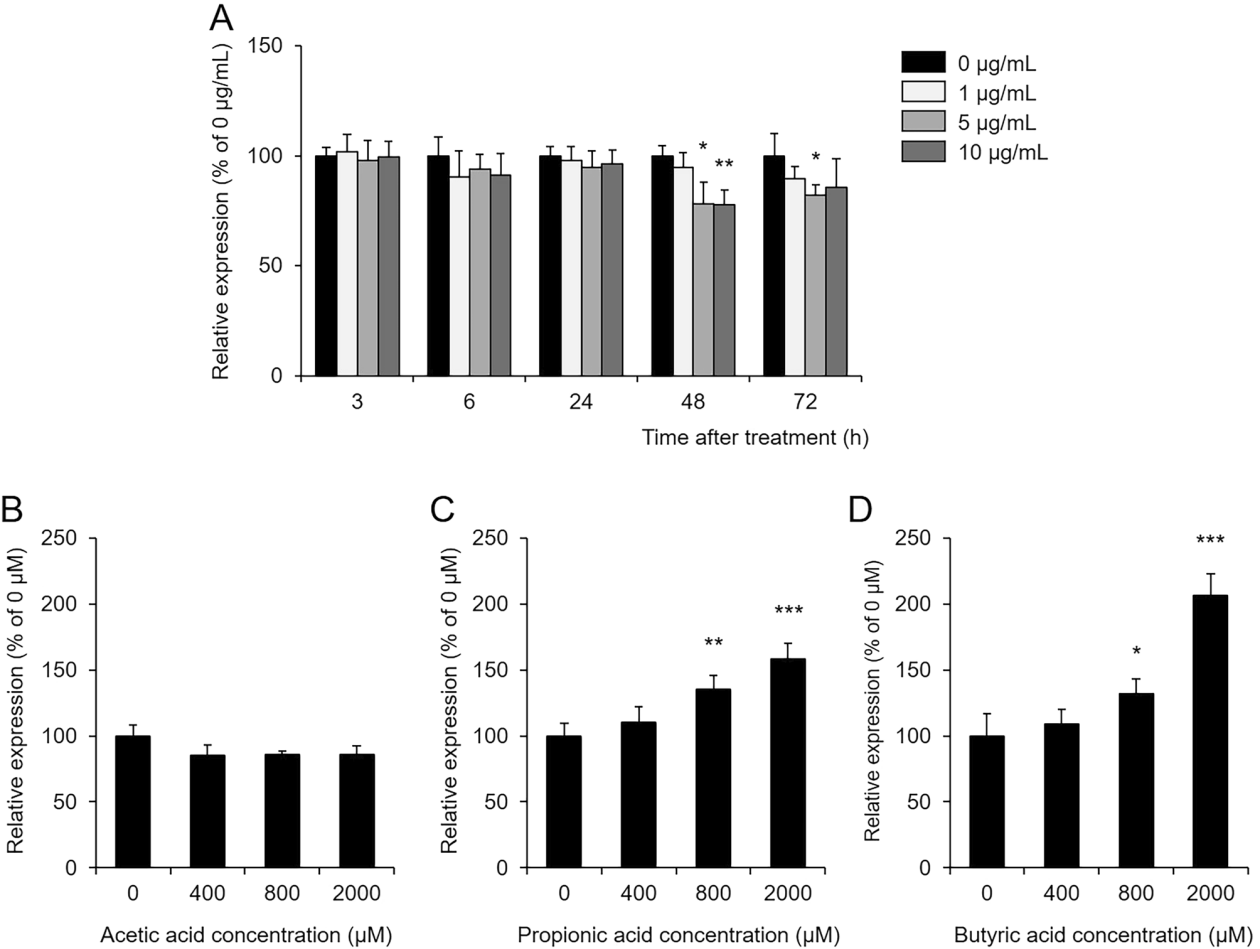
Effect of GTE and SCFAs on UGT1A1 expression in HT-29 cells. GTE (**A**), acetic acid (**B**), propionic acid (**C**), and butyric acid (**D**) were added to HT-29 cells, and the mRNA level of UGT1A1 was measured. UGT1A1 levels were normalized using GAPDH levels and are shown as 100% of the mean value in the control group (mean ± S.D., n = 4, *p < 0.05, **p < 0.01, ***p < 0.001 vs. 0 μg/mL or 0 μM).

We investigated whether GTE directly alters the expression of UGT1A1 in the colon using human colon cancer-derived HT-29 cells. As a result, even if GTE was added to HT-29 cells and cultured for up to 24 h, no change in the expression level of UGT1A1 was observed. When GTE was incubated at concentrations of 5 μg/mL or higher for 48 to 72 h, the UGT1A1 expression level was significantly lower in these cells than in the control cells (Fig. 4A). These results indicated that it was unlikely that GTE directly increased colonic UGT1A1 expression.

### Effects of short-chain fatty acid (SCFA) on the expression level of UGT1A1 in HT-29 cells

GTE and its constituent polyphenols have been shown to improve nonalcoholic fatty liver disease (NAFLD) and dextran sulfate sodium (DSS)-induced colitis via the gut microbiota^22,23^. It is also known that GTE increases the levels of SCFAs, which are metabolites derived from the gut microbiota^24^, and that SCFAs regulate the expression of UGT1A1^25^. Therefore, we investigated the possibility that GTE acted on the gut microbiota and increased the expression of UGT1A1 in the colon.

First, we confirmed whether SCFAs increase UGT1A1 expression in HT-29 cells. When acetic acid was added to HT-29 cells, the mRNA level of UGT1A1 did not change at any concentration (Fig. 4B). In contrast, propionic acid and butyric acid increased the UGT1A1 level in a dose-dependent manner (Fig. 4C,D).

**Figure 5:**
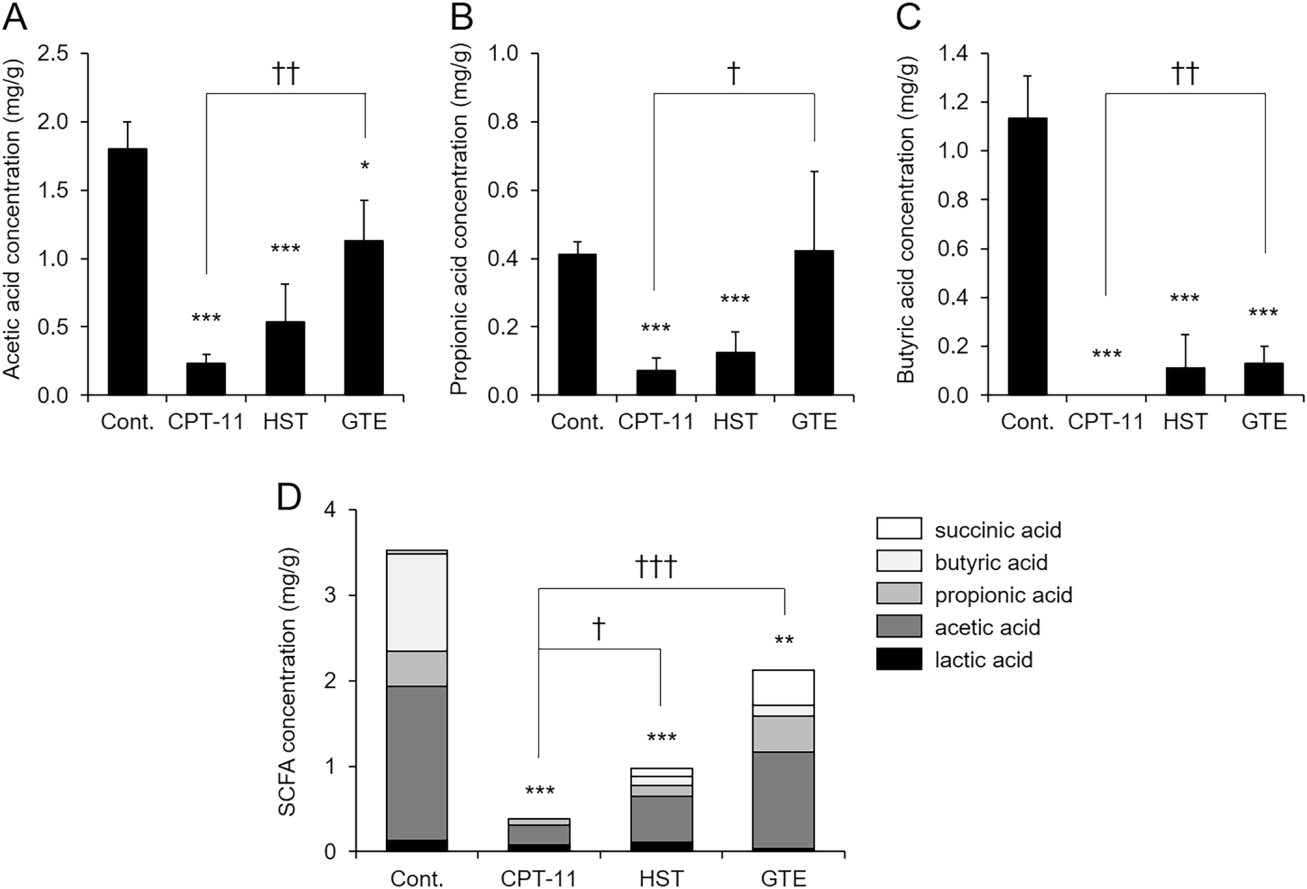
Changes in SCFA concentrations in the cecum after CPT-11 and GTE treatment. Rats were administered CPT-11 or lactic acid buffer intravenously for 4 days, and HST or GTE was administered in combination. Acetic acid (**A**), propionic acid (**B**), butyric acid (**C**), and total SCFA concentrations (**D**) in the cecum were measured by HPLC (mean ± S.D., n = 5, *p < 0.05, **p < 0.01, ***p < 0.001 vs. Cont., ^†^*p* < 0.05, ^††^*p* < 0.01, ^†††^*p* < 0.001 vs. CPT-11).

Next, we measured the concentration of SCFAs in the cecal contents of rats in which diarrhea was improved by the administration of GTE. Among the detected SCFAs, rats treated with CPT-11 showed significantly lower concentrations of acetic acid, propionic acid, and butyric acid than rats in the control group, and the total SCFA concentration decreased (Fig. 5). In contrast, GTE treatment significantly improved CPT-11-induced decreases in SCFA levels, and in particular, propionic acid levels recovered to the same level as that in the control group. The total concentration of SCFAs in the HST-treated group was higher than that in the CPT-11-treated group, but this effect was weaker than that in the GTE-treated group (*p* = 0.02 vs. GTE) (Fig. 5D). These results suggest that GTE may have increased the expression of colon UGT1A1 through increased production of SCFAs.

### Changes in the gut microbiota mediated by GTE treatment

GTE may have suppressed the CPT-11-induced decrease in colonic UGT1A1 expression by acting on the gut microbiota and increasing the levels of gut microbiota metabolites. Therefore, we analyzed the changes in the gut microbiota induced by GTE treatment using next-generation sequencing. In the CPT-11-treated group, the phyla *Firmicutes* and *Proteobacteria* accounted for approximately 30% of the total, followed by *Bacteroidetes*. The CPT-11-treated group exhibited a higher proportion of *Bacteroidetes* and *Proteobacteria* than the control group. In contrast, the phylum *Firmicutes* accounted for approximately 30% in the GTE-treated group, followed by *Bacteroidetes*, *Proteobacteria*, and *Verrucomicrobia*. GTE resulted in a lower proportion of *Proteobacteria* than CPT-11 alone but a higher proportion of *Verrucomicrobia*. In the HST-treated group, the proportion of the phylum *Firmicutes* was the highest, and the gut microbiota composition was similar to that in the control group (Table 1 and Supplemental Fig. 1).

**Table 1:** Phylum level proportions of gut microbiota.

Phylum	Cont.	CPT-11	HST	GTE
*Firmicutes*	42.7 ± 2.5	33.4 ± 4.9	33.4 ± 7.7	28.8 ± 3.2**
*Bacteroidetes*	3.2 ± 0.2	15.8 ± 4.7*	3.0 ± 1.4^†^	22.5 ± 0.8***
*Proteobacteria*	–	29.2 ± 10.8*	1.2 ± 0.6^†^	14.3 ± 6.8*
*Verrucomicrobia*	0.1 ± 0.1	2.0 ± 1.0	2.8 ± 1.8	10.0 ± 2.6*^,†^
*Actinobacteria*	0.4 ± 0.1	0.7 ± 0.1	0.9 ± 0.3	2.5 ± 0.9*^,†^
*Deferribacteres*	–	–	2.1 ± 0.8*^,†^	0.1 ± 0.0**^,†^
Reject hit	53.5 ± 2.3	18.9 ± 8.9	56.6 ± 8.7	21.8 ± 6.0

Rats were administered CPT-11 or lactic acid buffer intravenously for 4 days, and HST or GTE was administered in combination. The relative abundance at the phylum level in the gut microbiota was measured by next-generation sequencing. “–” indicates that the value is greater than 0 and less than 0.1 (mean ± S.D., n = 3, *p < 0.05, **p < 0.01, ***p < 0.001 vs. Cont., ^†^*p* < 0.05 vs. CPT-11).

**Figure 6:**
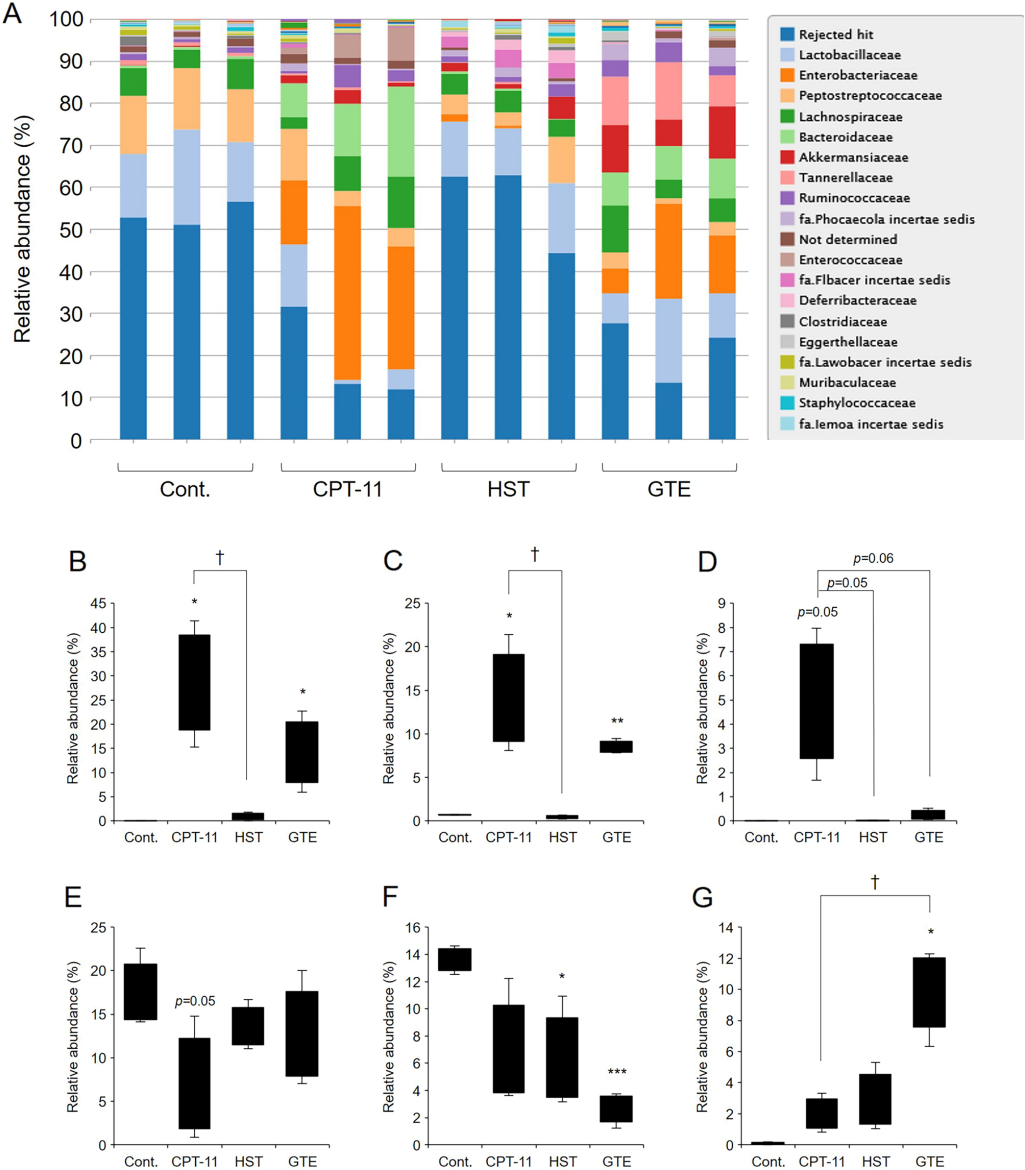
Relative abundance at the family level in the gut microbiota. Rats were administered CPT-11 or lactic acid buffer intravenously for 4 days, and HST or GTE was administered in combination. The relative abundance at the family level in the gut microbiota was measured by next-generation sequencing (**A**). Boxplots showing *Enterobacteriaceae* (**B**), *Bacteroidaceae* (**C**), *Enterococcaceae* (**D**), *Lactobacillaceae* (**E**), *Peptostreptococcaceae* (**F**), and *Akkermansiaceae* (**G**) of taxonomic abundance at the family level (mean ± S.D., n = 3, *p < 0.05, **p < 0.01, ***p < 0.001 vs. Cont., ^†^*p* < 0.05 vs. CPT-11).

Further analysis at the family level revealed that the CPT-11-treated group had a higher proportion of *Enterobacteriaceae*, *Bacteroidaceae*, and *Enterococcaceae* and a lower proportion of *Lactobacillaceae*. GTE treatment ameliorated the increased proportion of *Enterococcaceae* induced by CPT-11 treatment. A similar trend was observed in the HST-treated group (Fig. 6 and Table 2). These results indicated that GTE can alter gut microbiota induced by CPT-11.

**Table 2:** Family level proportions of gut microbiota.

Family	Cont.	CPT-11	HST	GTE
*Lactobacillaceae*	17.3 ± 3.8	6.8 ± 5.9	13.6 ± 2.3	12.5 ± 5.5
*Enterobacteriaceae*	–	28.7 ± 10.7*	0.9 ± 0.7^†^	14.2 ± 6.8*
*Peptostreptococcaceae*	13.6 ± 0.9	6.8 ± 3.9	6.2 ± 3.4*	2.7 ± 1.1***
*Lachnospiraceae*	6.1 ± 1.2	7.8 ± 3.9	4.7 ± 0.4	7.1 ± 3.0
*Bacteroidaceae*	0.7 ± 0.0	13.9 ± 5.5*	0.5 ± 0.2^†^	8.5 ± 0.7**
*Akkermansiaceae*	0.1 ± 0.1	2.0 ± 1.0	2.8 ± 1.8	10.0 ± 2.6*^, †^
*Tannerellaceae*	0.9 ± 0.2	0.5 ± 0.1*	0.2 ± 0.2*	10.9 ± 2.6*^, †^
*Ruminococcaceae*	1.2 ± 0.2	2.9 ± 1.9	1.9 ± 0.9	3.6 ± 1.0*
*fa.* *Phocaecola* *incertae* *sedis*	0.4 ± 0.0	0.7 ± 0.7	1.4 ± 0.6	3.0 ± 1.5
*Enterococcaceae*	–	5.0 ± 2.6	–	0.2 ± 0.2
*fa.* *Flbacer* *incertae* *sedis*	–	0.5 ± 0.3	3.6 ± 0.6**^, ††^	0.1 ± 0.1
*Deferribacteraceae*	–	–	2.1 ± 0.8*^, †^	0.1 ± 0.0**^, ††^
*Clostridiaceae*	1.0 ± 0.9	0.2 ± 0.1	0.7 ± 0.4	0.3 ± 0.2
*Eggerthellaceae*	0.2 ± 0.1	0.1 ± 0.0	0.5 ± 0.2^†^	1.2 ± 0.7
*fa.* *Lawobacer* *incertae* *sedis*	0.7 ± 0.5	0.3 ± 0.3	0.6 ± 0.5	0.2 ± 0.2
*Muribaculaceae*	0.7 ± 0.2	0.6 ± 0.3	0.5 ± 0.3	0.1 ± 0.0*
*Staphylococcaceae*	0.5 ± 0.3	0.1 ± 0.1	0.4 ± 0.4	0.5 ± 0.2
*fa.* *Iemoa* *incertae* *sedis*	0.1 ± 0.1	0.1 ± 0.1	1.1 ± 0.4*^, †^	–
*Bifidobacteriaceae*	–	0.4 ± 0.1*	0.2 ± 0.0*	0.5 ± 0.1*
*Rikenellaceae*	0.6 ± 0.2	0.1 ± 0.1*	0.3 ± 0.3	–
*Streptococcaceae*	0.1 ± 0.0	0.5 ± 0.2*	0.2 ± 0.1	0.2 ± 0.0*
*Coriobacteriaceae*	–	–	–	0.6 ± .2*^, †^
*Erysipelotrichaceae*	–	0.5 ± 0.5	–	–
*Micrococcaceae*	0.1 ± 0.0	0.1 ± 0.1	0.1 ± 0.1	0.2 ± 0.0
*Desulfovibrionaceae*	–	–	0.3 ± 0.2	0.1 ± 0.0
*Eubacteriaceae*	0.4 ± 0.1	–**	–**	–*
*Morganellaceae*	–	0.5 ± 0.4	–	–
*Aerococcaceae*	–	–	–	0.1 ± 0.1
*Corynebacteriaceae*	–	–	0.1 ± 0.1	–
Not determined	1.5 ± 0.2	1.9 ± 0.2	0.5 ± 0.3	1.2 ± 0.8
Reject hit	53.5 ± 2.3	18.9 ± 8.9	56.6 ± 8.7	21.8 ± 6.0

Rats were administered CPT-11 or lactic acid buffer intravenously for 4 days, and HST or GTE was administered in combination. The relative abundance at the family level in the gut microbiota was measured by next-generation sequencing. “–” indicates that the value is greater than 0 and less than 0.1 (mean ± S.D., n = 3, *p < 0.05, **p < 0.01, ***p < 0.001 vs. Cont., ^†^*p* < 0.05, ^††^*p* < 0.01 vs. CPT-11).

## Discussion

In this study, we focused on the use of GTE as a new strategy to prevent CPT-11-induced delayed diarrhea and verified its efficacy. When CPT-11 was administered intravenously to rats at a dose of 100 mg/kg/day for 4 days, body weight and 24-h food intake were significantly lower in these rats than in the control group (Fig. 1B,C). In addition, 72 h after the final administration of CPT-11, the fecal water content was approximately 3.5 times higher than that in the control group (Fig. 1D), indicating inflammation in the colon (Fig. 1E–G). These results were consistent with the characteristics of CPT-11-induced delayed diarrhea^16,26^. When GTE was orally administered to these rats 7 days before CPT-11 administration, the decrease in body weight and food intake by CPT-11 treatment was suppressed, and the increases in fecal water content and diarrhea were improved (Fig. 1B–D). In this study, since the periods of GTE and HST administration were different, direct comparison between the two groups was not possible. However, the antidiarrheal effect of GTE seems to be stronger than that of HST, which is currently used as a therapeutic agent. These results suggested that GTE could be an effective tool for preventing diarrhea caused by CPT-11.

Next, we examined the mechanism of the improvement of CPT-11-induced diarrhea mediated by GTE. β-glucuronidase is present in intestinal epithelial cells and immune cells such as macrophages, and β-glucuronidase derived from gut microbiota plays an important role in CPT-11-induced diarrhea^8^. Among several hundred types of β-glucuronidases derived from gut microbiota, β-glucuronidase encoding Loop1 exhibits high hydrolytic activity against SN-38G^17^. In this study, the abundances of *B.* *fragilis*, *E.* *coli*, *C.* *perfringens*, and *E.* *eligens*, which encode Loop1, were significantly higher in the CPT-11-treated group with diarrhea than in the control group. In contrast, GTE suppressed the increase in the abundances of these intestinal bacteria (Fig. 2C–F) and directly inhibited β-glucuronidase activity (Fig. 2A). GTE contains various polyphenols, such as epigallocatechin gallate (EGCG), epicatechin gallate (ECG), epigallocatechin (EGC), gallocatechin, and gallocatechin gallate. Among these polyphenols, EGCG and ECG strongly inhibit β-glucuronidase activity^15,27^, and EGCG also has antibacterial activity^28^. The GTE used in this study contains polyphenols such as EGCG, ECG, and EGC, and EGCG is the most abundant, containing 57%. These results suggest that polyphenols contained in GTE prevented CPT-11-induced colitis by directly inhibiting β-glucuronidase activity and by decreasing the levels of β-glucuronidase-producing bacteria.

CPT-11-induced delayed diarrhea is more severe in patients with UGT1A1 gene polymorphisms. In brief, it has been reported that patients with UGT1A1*27 or 1*28 have a 5.2-fold higher risk of developing severe diarrhea^18^. This is because SN-38 enters directly into the intestinal tract as a result of decreased UGT1A1 activity in the liver. Recently, it was reported that UGT1A1 is also abundantly expressed in the gastrointestinal tract and that CPT-11-induced gastrointestinal inflammation and mucosal damage were reduced in mice in which UGT1A1 was specifically expressed in the gastrointestinal tract than in mice in which UGT1A1 was specifically expressed in the liver^21^. In addition, CPT-11 administration to liver-specific UGT1A1-deficient mice resulted in the same mortality as that in wild-type mice, whereas CPT-11-induced diarrhea and intestinal mucositis were more severe in gut-specific UGT1A1-deficient mice than in wild-type mice^29,30^. These findings suggest that gastrointestinal UGT1A1 plays an important role in reducing SN-38 toxicity in the gut. Therefore, we focused on the function of gastrointestinal UGT1A1 as a mechanism by which GTE improves CPT-11-induced delayed diarrhea. The expression level of UGT1A1 in the group administered CPT-11 was only significantly lower than that in the control group in the colon. In contrast, GTE treatment suppressed this decrease (Fig. 3B,C). Since GTE did not directly increase UGT1A1 levels (Fig. 4A), GTE may have indirectly increased colonic UGT1A1 expression and improved CPT-11-induced delayed diarrhea.

To date, it has been reported that the expression level of intestinal UGT1A1 is regulated by SCFAs, which are intestinal bacterial metabolites^25^. It has also been reported that GTE increases the concentration of SCFAs in the intestine and that GTE and its constituent polyphenols affect the gut microbiota^22,24^. Therefore, we investigated the possibility that GTE acted on the gut microbiota and increased the expression of UGT1A1 in the colon. The results showed that among SCFAs, propionic acid and butyric acid increased UGT1A1 expression (Fig. 4C,D). The concentrations of propionic acid and butyric acid were higher in the GTE-treated group than in the group treated with CPT-11 alone (Fig. 5B,C). Propionic acid and butyric acid are mainly produced by intestinal bacteria such as *Bacteroides*, *Eubacterium*, and *Clostridium*, among which *Bacteroides* utilizes lactic acid in the intestine to produce propionic acid and succinic acid^31^. When GTE was administered, the levels of *Lactobacillaceae* that produced lactic acid and *Bacteroideaceae* were present in a constant ratio (Fig. 6C, E and Table 2), and the concentrations of propionic acid and succinic acid were high (Fig. 5D). Although the gut microbiota in the HST-treated group was similar to that of the control group, it is possible that the abundance of *Bacteroidaceae* was not sufficient to compensate for the decrease in propionic acid concentration due to CPT-11. It is also very interesting that the proportion of *Akkermansiaceae* increased in the GTE-treated group (Fig. 6G and Table 2). *Akkermansia* *muciniphila*, which belongs to *Akkermansiaceae*, is a mucin-producing bacterium, and it has been reported that the levels of sulfated mucins and the abundance of *A.* *muciniphila* were decreased in patients with ulcerative colitis^32^. It has also been reported that administration of *A.* *muciniphila* improves DSS-induced colitis^33,34^. Therefore, the effect of GTE on *Akkermansiaceae* may also help to suppress CPT-11-induced diarrhea.

The expression of UGT1A1 is regulated by the nuclear receptors aryl hydrocarbon receptor (AhR) and pregnane X receptor, and propionic acid and butyric acid have strong ligand activity for AhR^25^. We also found that treatment of HT-29 cells with propionic acid and butyric acid increased the expression of AhR target genes (Supplemental Fig. 2), indicating that propionic acid and butyric acid activated AhR. Therefore, it was considered that propionic acid and butyric acid increased the expression of colon UGT1A1 by acting on AhR.

This study also revealed that GTE inhibited the decrease in food intake caused by CPT-11. This effect was already observed before the onset of CPT-11-induced diarrhea, that is, 24 h after the final administration (Fig. 1C,D). Since there was no difference in the food intake between rats treated with GTE alone and those in the control group (data not shown), GTE itself does not seem to increase food intake. Although the detailed mechanism by which the amount of food intake was maintained in the GTE-treated group is unknown, the proliferation and differentiation of colonic epithelial cells are regulated by feeding^35,36^. Therefore, the effect of GTE on maintaining food intake may contribute to the improvement of intestinal mucosal disorders in addition to suppressing body weight loss caused by CPT-11.

In conclusion, GTE prevents the development of CPT-11-induced colitis. It is also suggested that this mechanism involves inhibition of β-glucuronidase activity, a decrease in the abundance of β-glucuronidase-producing bacteria, and suppression of SN-38 production associated with an increase in colonic UGT1A1 levels by regulating the gut microbiota. In addition, GTE suppressed anorexia and weight loss caused by CPT-11. To date, it has been reported that GTE improves CPT-11-induced oral mucositis^37^. Therefore, taking GTE as a beverage or supplement during CPT-11 use may be a useful tool to reduce the adverse effects caused by CPT-11 and may contribute to improving the QOL of cancer patients.

## Materials and methods

### Materials

CPT-11 hydrochloride was purchased from Carbosynth Limited (Berkshire, UK). HST extract powder (Lot. No. 2120014010) was purchased from Tsumura and Co. (Tokyo, Japan). Teafuran 90S (Lot. No. 201110-2, Ito En Ltd., Tokyo, Japan) was used as the GTE. Teafuran 90S is a dry powder made by extracting tea leaves with hot water, followed by purification with adsorption columns and ethanol, and the total polyphenol content is more than 90%.

### Experimental design and assessment of diarrhea

Wistar rats (6 weeks old, male) were purchased from Japan SLC (Shizuoka, Japan) and housed at a temperature of 24 ± 1 °C and a humidity of 55 ± 5%. After being bred for a week, the animals were used for experiments.

The CPT-11-induced diarrhea rat model was established by a previously reported method^26^. A total of 20 rats were divided into four groups: control group, CPT-11-treated group, HST-treated group, and GTE-treated group (n = 5 per group). Rats were administered lactic acid buffer (45 mg/mL d-sorbitol, 0.9 mg/mL lactic acid; pH 3.4) or CPT-11 (100 mg/kg/day) intravenously for 4 days. In the control group, distilled water was orally administered twice a day for 14 days starting 7 days before administration of lactic acid buffer. In the CPT-11-treated group, distilled water was orally administered twice a day for 14 days starting 7 days before administration of CPT-11. In the HST-treated group, HST (1000 mg/kg/day) was orally administered twice a day for 8 days starting 1 day before administration of CPT-11. In the GTE-treated group, GTE (1000 mg/kg/day) was orally administered twice a day for 14 days starting 7 days before the administration of CPT-11 (Fig. 1A). Three days after the last administration of CPT-11, the small intestine, large intestine, and intestinal contents were collected under isoflurane anesthesia and stored at −80 °C. At 24 h, 48 h, and 72 h after the final administration of CPT-11, excreted feces were collected and dried with an FDM-1000 freeze dryer (EYELA, Tokyo, Japan), and fecal water content (%) was calculated.

### Cell culture

Human colon cancer-derived HT-29 cells were maintained in Roswell Park Memorial Institute (RPMI) 1640 medium containing 100 U/mL penicillin G potassium, 100 μg/mL streptomycin, 0.25 μg/mL amphotericin B, and 10% fetal bovine serum. Cells were seeded in 24-well plates, and subconfluent cells were treated with GTE (0–10 μg/mL), acetic acid (0–2000 μM), propionic acid (0–2000 μM), or butyric acid (0–2000 μM) for 3, 6, 24, 48, or 72 h.

### Real-time PCR

Total RNA was extracted from small intestine, large intestine, or HT-29 cells using TRI Reagent (Sigma-Aldrich Corp., St. Louis, MO, USA), and cDNA was synthesized using a High Capacity cDNA Synthesis Kit (Applied Biosystems, Foster City, CA, USA). The primers shown in Supplemental Table 1 were prepared, and the expression level of each gene was measured by a CFX Connect Real-Time PCR System (Bio-Rad Laboratories, Hercules, CA, USA) under the following conditions: denaturation at 95 °C for 15 s, annealing at 56 °C for 30 s, and elongation at 72 °C for 30 s.

### Western blotting

The mucosa was scraped from the rat colon, suspended in dissecting buffer (0.3 M sucrose, 25 mM imidazole, 1 mM ethylenediaminetetraacetic acid; pH 7.2) and homogenized on ice. The homogenate was centrifuged (800×*g*, 4 °C, 15 min), and the supernatant was further centrifuged (200,000×*g*, 4 °C, 60 min). The supernatant was removed, and the precipitate was dispersed in dissecting buffer using an ultrasonic homogenizer (UH-50, SMT Co., Ltd, Tokyo, Japan) to prepare a sample solution.

After measuring the protein concentration of the sample solution, the solution was mixed with loading buffer. After electrophoresis of the sample solution on a polyacrylamide gel, it was transferred to polyvinylidene difluoride membranes. The membranes were treated with rabbit anti-UGT1A1 antibody (Abcam, Cambridge, UK) or rabbit anti-β-actin antibody (Bio regend, San Diego, CA, USA) as the primary antibody and donkey anti-rabbit IgG-HRP antibody (Cytiva, Tokyo, Japan) as the secondary antibody. After the membrane was washed, it was treated with ECL prime Western blotting detection reagents (Cytiva), and the bands detected by Image Quant LAS500 (Cytiva) were analyzed.

### β-glucuronidase activity assay

The enzyme solution was obtained by homogenizing normal rat feces with a microhomogenizer and centrifuging (10,000×*g*, 5 min, 4 °C). The enzyme solution was mixed with HST or GTE (final concentration: 0–400 μg/mL), and the β-glucuronidase substrate solution was added and incubated at 37 °C for 60 min. The fluorescence signal at the excitation wavelength of 365 nm and emission wavelength of 415–445 nm were measured by a GloMax Discover Microplate Reader (Promega, Madison, WI, USA).

### Measurement of SCFA levels

The cecal contents were heat-treated (85 °C, 15 min) after suspension in the extraction solution. After the homogenates were crushed with beads, the samples were centrifuged (18,400×*g*, 10 min), and the supernatant was filtered through a membrane filter (0.20 μm) to obtain a sample solution. The concentrations of SCFAs were measured using high-performance liquid chromatography.

### Quantification of bacteria from rat feces

Extraction of bacterial DNA from rat feces was performed using the QIAamp Fast DNA Stool Mini Kit (Qiagen, Valencia, CA, USA). The primers shown in Supplemental Table 2 were prepared, and the levels of each gut bacterium were measured using the CFX Connect Real-Time PCR System (Bio-Rad Laboratories) under the following conditions: denaturation at 95 °C for 30 s, annealing at 58 °C for 30 s, and elongation at 72 °C for 60 s.

### Gut microbiota data analysis

Homogenization of rat feces was performed according to a previously described method^38^. Then, DNA was extracted using an automated DNA isolation system (GENE PREP STAR PI-480 KURABO, Tokyo, Japan). The V3–V4 regions of bacterial and archaeal 16S rRNA were amplified using the Pro341F/Pro805R primers and the dual-index method^39^. Barcoded amplicons were paired-end sequenced on a 2 × 301-bp cycle using the MiSeq system with MiSeq Reagent Kit version 3 (600 Cycle) chemistry. The primer sequences on paired-end sequencing reads were trimmed by Cutadapt ver 1.18 with default settings. Paired-end sequencing reads were merged using the fastq-join program with default settings. Only joined reads that had quality value scores of ≥ 20 for more than 99% of the sequences were extracted using FASTX-Toolkit. The chimeric sequences were deleted with usearch61. Nonchimeric reads were submitted for 16S rDNA-based taxonomic analysis using the Ribosomal Database Project ver 2.13 (RDP) and the TechnoSuruga Lab Microbial Identification database ver 16.0 (DB-BA, TechnoSuruga Laboratory, Shizuoka, Japan) with homology ≥ 97%^40,41^.

### Statistical analysis

Experimental values are expressed as the mean ± standard deviation (SD). Dunnett’s test of multiple comparison was used for statistical significance. Tukey’s test was performed after one-way ANOVA for multiple comparisons of three or more groups. Statistical significance was considered to exist when the risk rate was less than 5% (*p* < 0.05).

### Ethical approval

The research reported in this study involved rats. This animal experiment was conducted with approval and in accordance with the Hoshi University Guiding Principles for the Care and Use of Laboratory Animals (approval number: 19-109). This study is reported in accordance with ARRIVE guidelines.

## Supplementary Information

Supplementary Information.

## Data Availability

All data generated or analyzed during this study are included in this published article and its supplementary information files.
