# Supplementary material for: Green tea extract prevents CPT-11-induced diarrhea by regulating the gut microbiota
Source: Sci Rep. 2023 Apr 21;13:6537. doi: 10.1038/s41598-023-33731-w (PMC10121581; doi:10.1038/s41598-023-33731-w)
Supplement: Supplementary file 1 — Supplementary Information. [file 41598_2023_33731_MOESM1_ESM.docx]

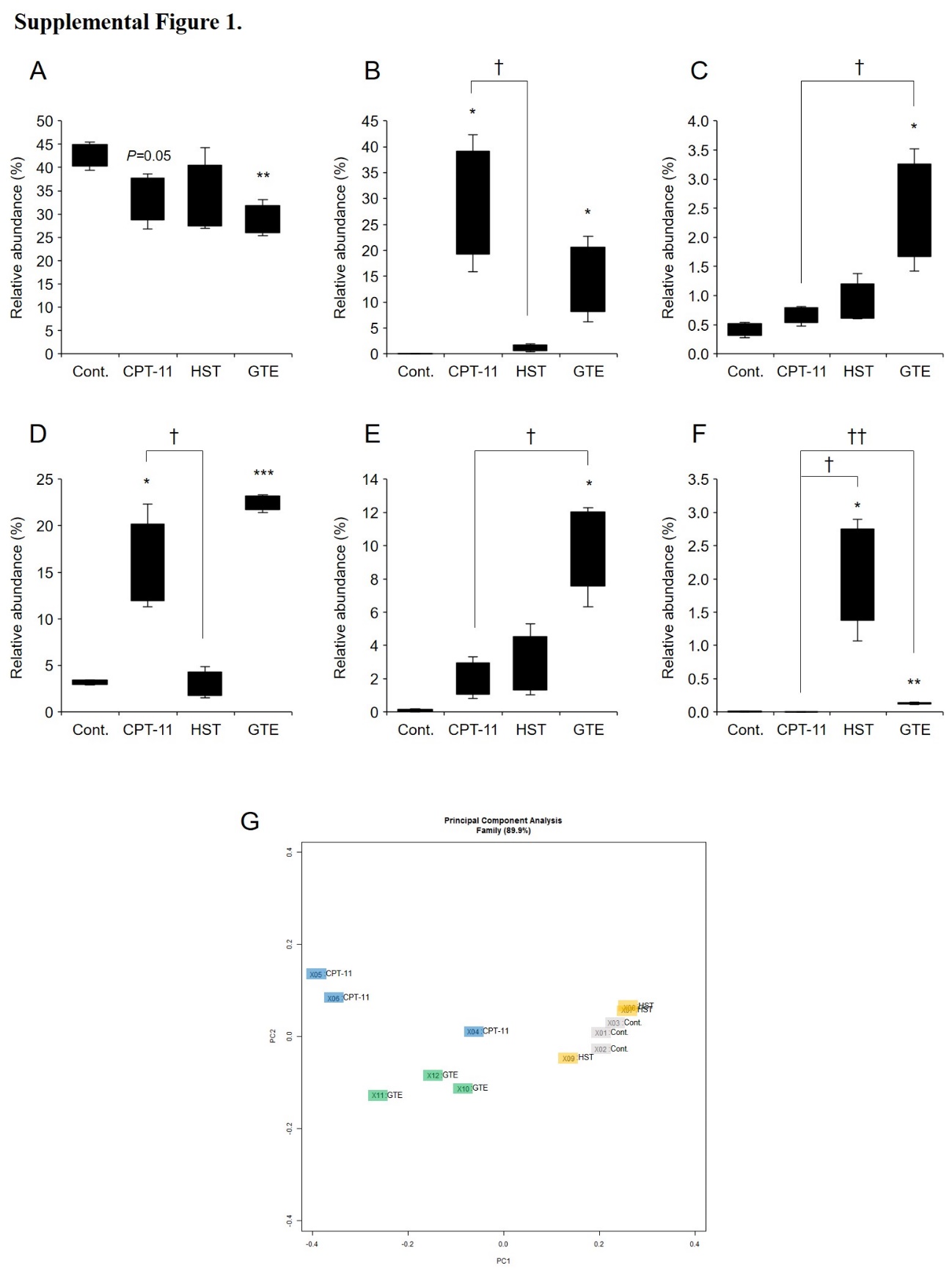


**Supplemental Figure 1. Relative abundance and similarity in the gut microbiota**

Rats were administered CPT-11 or lactic acid buffer intravenously for 4 days, and HST or GTE was administered in combination. The relative abundance in the gut microbiota was measured by next-generation sequencing. Boxplots showing *Firmicutes* (A), *Proteobacteria* (B), *Actinobacteria* (C), *Bacteroidetes* (D), *Verrucomicrobia* (E), and *Deferribacteres* (F) of taxonomic abundance at the phylum level (mean ± S.D., n＝3, **p* < 0.05, ***p* < 0.01, ****p* < 0.001 vs. Cont., ^†^*p* < 0.05, ^††^*p* < 0.01 vs. CPT-11). Principal coordinate analysis (PCoA) plot showing the similarity among rat microbiota compositions at family level (G).


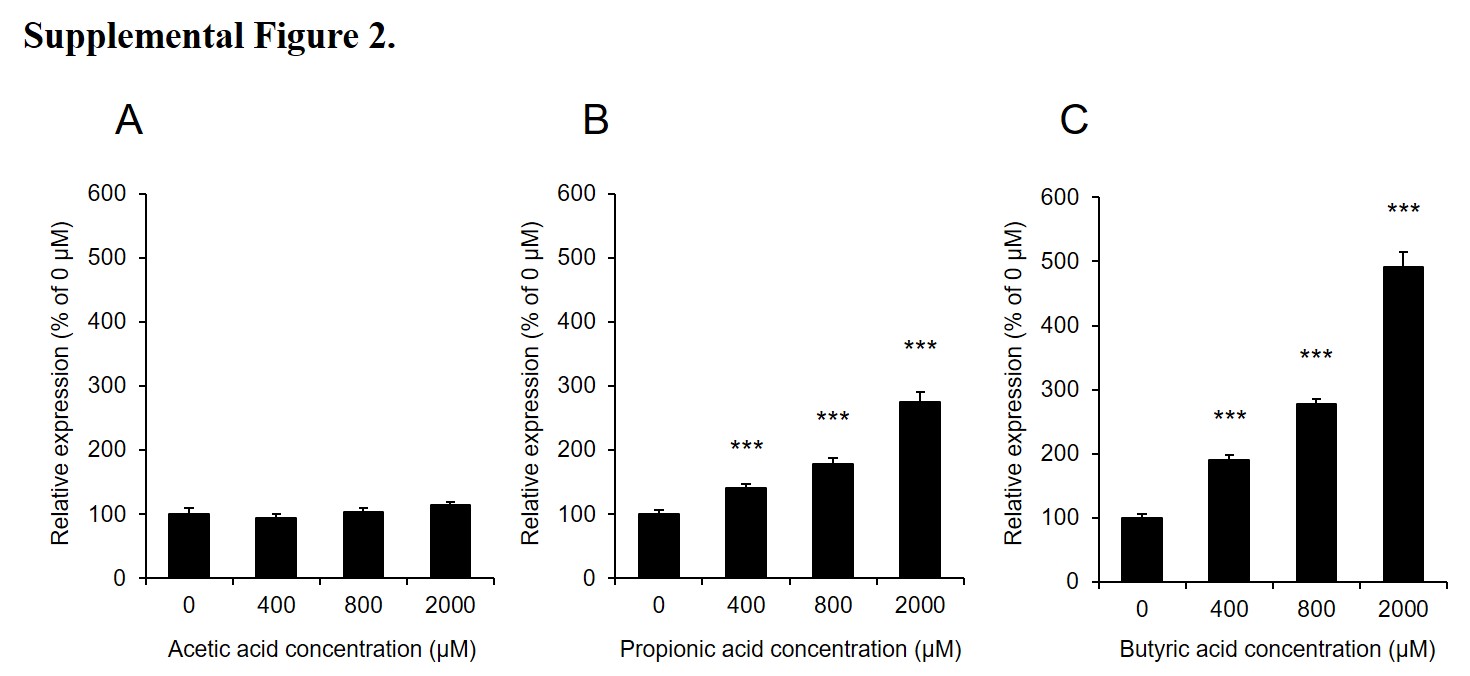


**Supplemental Figure 2. Effect of SCFAs on AhR expression in HT-29 cells**

Acetic acid (A), propionic acid (B), and butyric acid (C) were added to HT-29 cells for 24 h, and the mRNA expression level of AhR was analyzed. AhR levels were normalized using GAPDH levels and are shown as 100% of the mean value in the control group (mean ± S.D., n＝4, **p* < 0.05, ***p* < 0.01, ****p* < 0.001 vs. 0 μM).


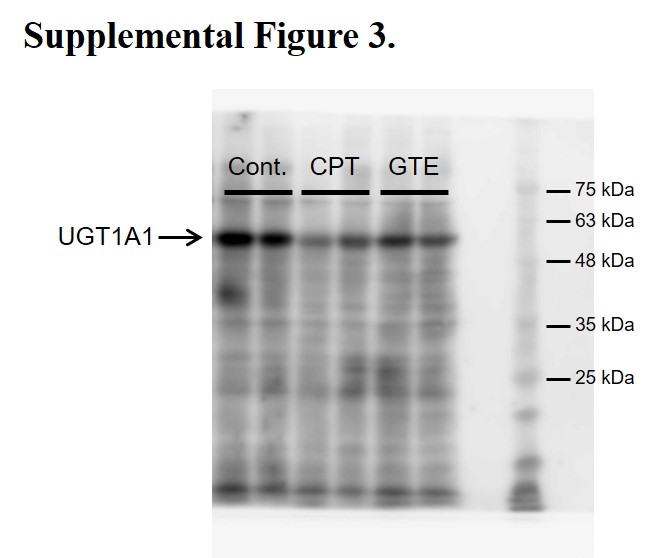


**Supplemental Figure 3. Full length of western blot for UGT1A1 protein expression**

Rats were administered CPT-11 or lactic acid buffer intravenously for 4 days, and HST or GTE was administered in combination. The protein expression levels of UGT1A1 in the colon was analyzed by Western blotting.

**Supplemental Table 1. Primer sequences**

| Gene | Forward (5’-3’) | Reverse (5’-3’) |
| --- | --- | --- |
| rIL-1β | TCAGGCTTCCTTGTGCAAGTGT | ACAGGTCATTCTCCTCACTGTC |
| rIL-6 | TAGTCCTTCCTACCCCAACTTC | GCCGAGTAGACCTCATAGTGAC |
| riNOS | CAAGCACATTTGGCAATGGA | GCCAAATACCGCATACCTGA |
| rUGT1a1 | TTACTCCCCCTATGGGTCAC | TCAAATTCCTGGGATAGGGC |
| rβ-actin | GCCACTGCCGCATCCTCTTG | CGGAACCGCTCATTGCCGAT |
| hUGT1a1 | CATGCTGGGAAGATACTGTTGAT | GCCCGAGACTAACAAAAGACTCT |
| hGAPDH | ATGGGGAAGGTGAAGGTGG | GGGGTCATTGATGGCAACAATA |

**Supplemental Table 2. Primer sequences for bacteria**

| Target | Forward (5’-3’) | Reverse (5’-3’) |
| --- | --- | --- |
| *Bacteroides fragilis* | ATAGCCTTTCGAAAGRAAGAT | CCAGTATCAACTGCAATTTTA |
| *Escherichia coli* | CAGTTGTCTCAGTTCATGGACC | ACCGATGTTTGGACCTTCAG |
| *Clostridium perfringens* | CGCATAACGTTGAAAGATGG | CCTTGGTAGGCCGTTACCC |
| *Eubacterium eligens* | CGCACAATGTTGCATGACAT | CTTAGTCAGGTACCGTCACTA |
| 16S rRNA | TCCTACGGGAGGCAGCAGT | GGACTACCAGGGTATCTAATCCTGTT |
